# Global Sensitivity Analysis of Chosen Harmonic Drive Parameters Affecting Its Lost Motion

**DOI:** 10.3390/ma14175057

**Published:** 2021-09-03

**Authors:** Slavomir Hrcek, Frantisek Brumercik, Lukas Smetanka, Michal Lukac, Branislav Patin, Adam Glowacz

**Affiliations:** 1 Department of Design and Mechanical Elements, Faculty of Mechanical Engineering, University of Zilina, 010 26 Zilina, Slovakia; slavomir.hrcek@fstroj.uniza.sk (S.H.); lukas.smetanka@fstroj.uniza.sk (L.S.); michal.lukac@fstroj.uniza.sk (M.L.); branislav.patin@fstroj.uniza.sk (B.P.); 2 Department of Automatic Control and Robotics, Faculty of Electrical Engineering, Automatics, Computer Science and Biomedical Engineering, AGH University of Science and Technology, 30-059 Krakow, Poland; adglow@agh.edu.pl

**Keywords:** harmonic drive, lost motion, torsional backlash, FEA analysis, global sensitivity analysis, Sobol indices

## Abstract

The aim of the presented study was to perform a global sensitivity analysis of various design parameters affecting the lost motion of the harmonic drive. A detailed virtual model of a harmonic drive was developed, including the wave generator, the flexible ball bearing, the flexible spline and the circular spline. Finite element analyses were performed to observe which parameter from the harmonic drive geometry parameter group affects the lost motion value most. The analyses were carried out using 4% of the rated harmonic drive output torque by the locked wave generator and fixed circular spline according the requirements for the high accuracy harmonic drive units. The described approach was applied to two harmonic drive units with the same ratio, but various dimensions and rated power were used to generalize and interpret the global sensitivity analysis results properly. The most important variable was for both harmonic drives the offset from the nominal tooth shape.

## 1. Introduction

Harmonic drive gearing, which is also called strain wave gearing, is a very progressive transmission with broad spectrum of use in applications with a high demand for precise positioning, e.g., aerospace, robot arms, test devices, medical equipment, etc. Harmonic drive (HD) consists of three main components: the wave generator (WG), the flexible spline (FS) and the circular spline (CS). One of the main parameters, which influences the selection and application of the HD, is the lost motion value, also called as torsional backlash.

Backlash is the amount by which the width of a gear’s tooth space exceeds the thickness of an engaging tooth measured at the pitch circle of the gears, whereas the transmission lost motion is a measurement of the angle of movement in both directions before movement at the input occurs while applying a load at the output. Backlash, also known as slop, lash, free-play, or just play, is an angular quantity caused by the circular geometry of the gear. This is known as clearance backlash, and it is required to account for manufacturing defects, providing space for lubrication, and allowing for component thermal expansion. The HDs are usually designed with zero backlash at the gear mesh.

**Figure 1:**
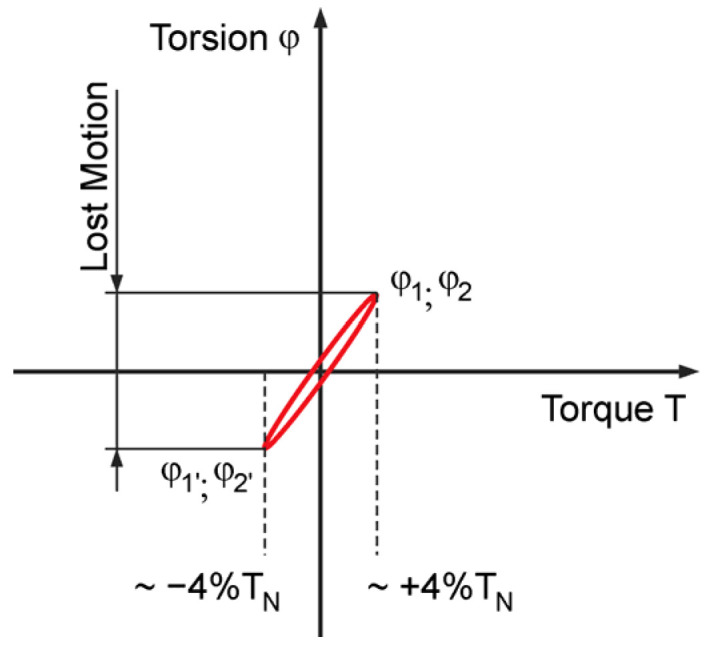
HD lost motion diagram.

The producers of high accuracy HD units guarantee various values of the maximum torsion *φ* during their operation life measuring the angular lost motion of the FS by low output torque values. The test is usually performed by applying the 4% of rated HD output torque *T_N_* to the FS and measuring its angular deformation *φ*_1_, *φ*_1_′ (*φ*_2_, *φ*_2_′, respectively) in both directions, with the WG locked and the CS fixed, obtaining the hysteresis curve shown in Figure 1.

The main goal of this article is to present a study that is focused on the global sensitivity analysis of the variable HD component dimensions influencing the lost motion value Φ of described HD units defined as

(1) Φ=φ+φ′

where:

*φ*—angular deformation by applying the +4% of *T_N_* to the FS;
*φ*′—angular deformation by applying the −4% of *T_N_* to the FS.

The lost motion of the HDs with various chosen component parameters is obtained by simulating the lost motion test procedure using the finite elements analysis (FEA) and processing the acquired data by appropriate mathematical methods.

An example of a dynamic model of the HD in a toothed transmission system was presented by Folega [1]. He discussed certain aspects of dynamic modeling of the harmonic drive and proposed a new original dynamic model of a harmonic drive for a power transmission system. His model takes into account the nonlinear stiffness changes and also the damping.

The approach by particular HD components modeling can vary according their complexity. The early original wave generator outer shape was an elliptoid described by Musser [2]. Further research led to the configuration curve defined by a four-term Fourier expansion equation defined by Ishikawa [3]. Gravagno et al. [4] discussed and quantitatively evaluated the influence of the wave generator shape on the pure kinematic error of HD. The Résal curve, an ellipse and two conjugate arcs curves yield the lowest kinematic error. The cam curves yield slightly higher kinematic error as a compromise including also a caterpillar effect at the poles of the WG.

There are also various teeth splines used by the common produced harmonic drives. The harmonic drive company uses S-shaped teeth with the geometry based on the patent of Ishikawa [5]. This tooth profile allows for up to 30% of the total number of teeth to be engaged at the same time. In addition, when compared to an involute tooth, the big tooth root radius boosts dental strength. This technological innovation produces high torque, high torsional rigidity, extended life, and smooth rotating as also described in [6]. The teeth shape optimization is presented, e.g., by Kayabashi [7], Kiyosawa et al. [8] and also in further patents of Ishikawa. The parametric design of double-circular-arc tooth profile and its influence on the functional backlash of harmonic drive was already described by Chen et al. [9]. They used the envelope theory to design a double circular arc tooth profile of rigid gear to achieve multi-section conjugation. The presented method is more consistent with the actual working condition of the harmonic gear compared with the method of only designing the tooth profile of the middle section conjugate. The geometry of the FS influences the torsional stiffness of the HD significantly.

The problem of the torsional stiffness affecting also the harmonic drive backlash value was analyzed in various references. Yang et al. [10] observed the backlash of the harmonic drive based on a parametric solid finite element model. They calculated the backlashes with the minimum circumferential distance between engaged involute tooth profiles along tooth height direction using the four-rollers wave generator. They observed that the validity of the backlash computation based on the FEM is higher by the contrastive analysis. Rhéaume et al. [11] presented a finite-element model to reproduce the behavior of the torsional stiffness of a harmonic drive, which allowed the evaluation of the effects of various geometrical parameters on the torsional stiffness.

Wang et al. [12] presented the measurement and analysis of backlash on harmonic drives, where the backlash of harmonic drive transmission is split into two primary components: clearance-induced lost motion and elastic deformation-induced lost motion. The tooth clearance between circular spline and flexspline, as well as the clearance from Oldham coupling in the wave generator, are the main sources of clearance, whereas the elastic deformation is primarily attributable to flexspline under torque load. Zou et al. [13] described the measurement and modeling of kinematic error and clearance in harmonic drive. They established an experimental setup to obtain the kinematic error and clearance in harmonic drive. The position-dependent kinematic error was modeled by the Fourier expression method.

The general global sensitivity analysis (GSA) approach is described in detail, e.g., by Saltelli et al. [14]. Many references deal with GSA of various gearbox components. Woll et al. [15] carried out a sensitivity analysis on the drivetrain of an offshore winch with active heave compensation. By adjusting the parameters within usual ranges and assessing the quantitative effect on the lifetime, the effects of the parameters’ gear center distance and ambient temperature were explored. Li et al. [16] performed a sensitivity analysis on a synchronization mechanism for manual transmission gearbox using parameterized virtual models of the typical synchronizers, and the paper discusses the influence of the main parameters of synchronizer on the gear shift performance. A sensitivity analysis for the proper tolerance choice of gear profiles was carried out by Rajabalinejad [17]. He explained how to use a sensitivity chart to identify the acceptable and ideal tolerance fields for profile modification as a tool for assessing the influence of profile alterations on the load factor of gears with stochastic indexing faults and finding optimal solutions. Ostapski [18] analyzed the harmonic drive wave generator-flexspline system in relation to selected structural parameters and manufacturing deviations. Material research, as well as simulations of the stress state in the bearing versus manufacturing deviations, and fits between the bearing and the generator cam were used to describe the problem of failure of the elastic bearing supporting the generator in a harmonic drive.

## 2. Materials and Methods

The lost motion (torsional backlash) is a key parameter defining the accuracy of the HD. When subjected to the rated torque, HD display characteristics shown in the hysteresis curve. When a torque is applied to the output shaft of the gear with the input shaft locked, the torque–torsion relationship can be measured at the output.

The lost motion is the term used to characterize the HD torsional stiffness in the low torque region. Lost motion is evaluated by a test applying the ±4% of rated HD output torque *T_N_* to the FS measuring its angular deformation in both directions with the WG locked and the CS fixed to obtain the torsional windup of the gear.

The standard kinematic gear ratio *i* of the HD is defined by the WG acting as the input shaft with the angular velocity *ω*_1_ (revolutions per minute *n*_1_), the FS with the teeth number *z_FS_* (−) acting as the output shaft with the angular velocity *ω*_2_ (revolutions per minute *n*_2_) and the CS with the teeth number *z_CS_* (−) fixed according the equation:

(2) $$i=\frac{\omega_{1}}{\omega_{2}}=\frac{n_{1}}{n_{2}}=-\frac{z_{FS}}{z_{CS}-z_{FS}}$$

The nominal parameters of harmonic drive units, which are analyzed in the presented research and denoted as HD 42 and HD 120 according the bearing outer diameter (flexspline inner diameter), are listed in Table 1.

The main goal of the presented research was to identify the most important parameters picked up from a parametric dimension set defining the HD geometry with the maximum influence to the lost motion value, and subsequently to the overall torsional backlash value by various motion states of the HD unit. The goal is to obtain the lost motion value Φ of maximum 1 arcmin alternating the identified most important HD unit parameters by next development including HD key components design changes.

The complex parametric modeling of the virtual tested HD units was carried out with detailed geometry of the WG, the ball bearing with flexible rings and the FS and CS with teeth splines. The set of the parameters influencing the backlash value was picked up. The research process was then executed in the following steps:

1. the parametric model of the HD finite element analyses was built and the analyses of the prescribed motion state were performed to determine the lost motion values for the generated set of chosen variables;
2. the correlation of the input parameters (flexible ball bearing inner and outer ring diameter, the radial bearing clearance and the offset of the FS and CS nominal teeth spline) and the output parameter (lost motion) was observed and the correlation coefficients were calculated;
3. the global sensitivity analyses (GSA) of the chosen variable sets were performed based on the obtained dependence of the input and output variables calculating the GSA indices according the generated quasi-random variable sets.

The calculation routine was performed for both HD units with various nominal power value to support the generalization of the calculation results, which were interpreted in the conclusion.

## 3. Parametric Model Building

**Figure 2:**
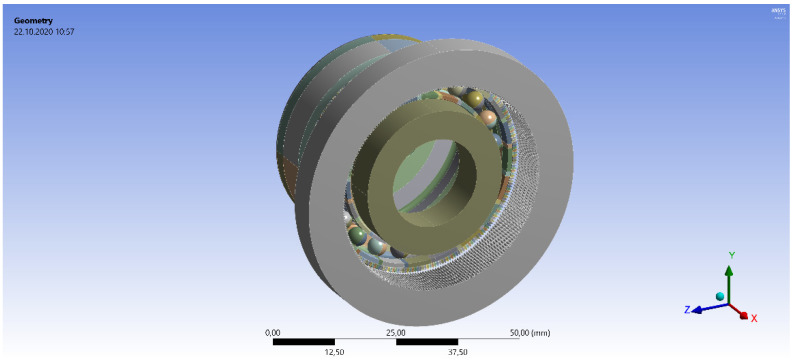
Model of the harmonic drive geometry of the HD 42 unit.

The parametric model of the HD was created in the Creo parametric software (PTC, Inc., Boston, MA, USA, 3.0) according the equations describing the variable component parameters generated in the Matlab software (Mathworks, Inc., Natick, MA, USA, 2018R2) as described by Majchrak et al. [19]. The prepared geometrical models were imported to the analysis software Ansys Workbench (Ansys, Inc., Canonsburg, PA, USA, 17.2) and processed there. There was a calculation routine built, which provided the automatic HD parametric model change according the needs of the FEA calculations. The complete parametric model of the examined HD 42 unit is presented in Figure 2.

The tested HD wave generator (WG) actual outer shape radius *R* is defined by the cam major axis *A*, cam minor axis *B* and the exponent *n* for the angle 0 ≤ *φ* ≤ π/2 by the equation:

(3) $$R=A+B\;\cos\;2\left(\frac{\pi }{2}{\left(\frac{\varphi }{\frac{\pi \;}{2}}\right)}^{n}\right)$$

**Figure 3:**
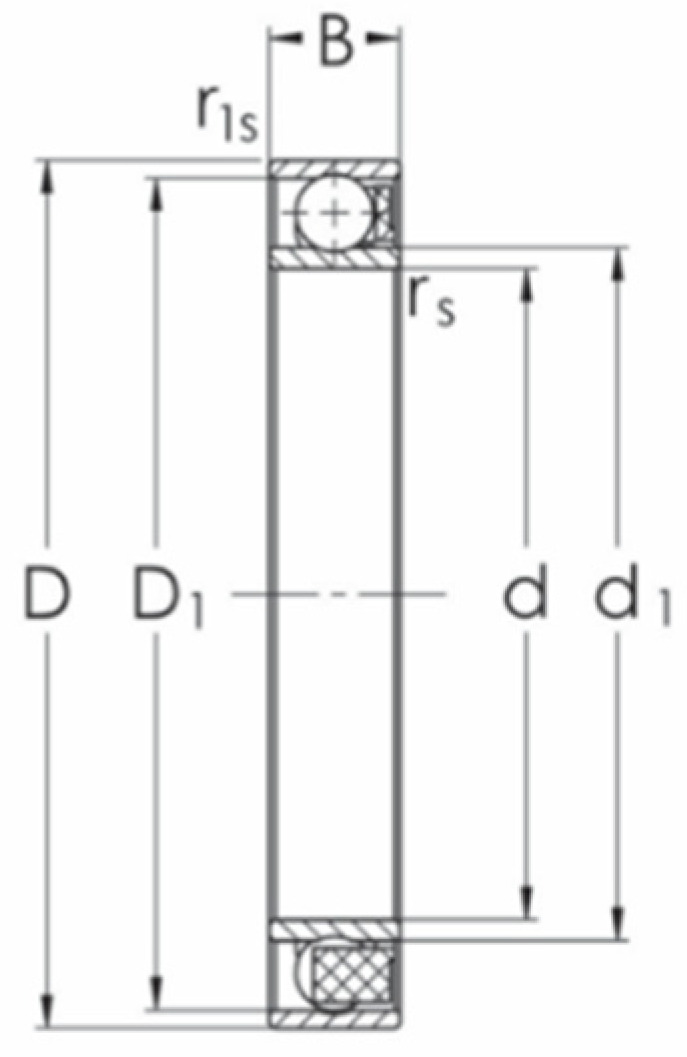
Ball bearing with flexible rings characteristic dimensions: inner ring inner diameter *d*; inner ring outer diameter *d*_1_; outer ring inner diameter *D*_1_; outer ring outer diameter *D*; bearing width *B*.

The variables defined in the WG outer shape equation influence the circumference of the WG and hence the inner ring inner diameter of the assembled ball bearing. The model of the ball bearing with flexible rings located on the WG outer shape is created by the method described by Steininger et al. [20]. The bearings’ measures are based on standard products of the PBF Krasnik factory (type 113-1145TN for the HD 42 and type 114-876TN for the HD 120) with characteristic dimensions shown in Figure 3 and the nominal dimensions listed in Table 2 [21].

The nominal radial internal clearance (ISO clearance class) and the ovalization of the inner ring influencing the range of diameters used by the analyses, is defined by the equation:

(4) $$O=\frac{d_{\max}-d_{\min}}{2}$$

where:

*d*_max_—maximum permissible value of the inner bearing diameter *d*;
*d*_min_—minimum permissible value of the inner bearing diameter *d*.

The standard bearings of the selected producer do not meet the required radial internal clearance values. The parametric model of the bearing is therefore defined as a custom bearing with standard nominal dimensions and lower value of the radial internal clearance, securing a better match of the bearing inner flexible ring to the shape of the WG without the occurrence of the undesirable blank areas in the bearing–WG surface contact area. The ball bearing in the presented research is, however, modeled without the physical separator between the rolling elements. The spacing between the balls is secured by the boundary conditions defined in the finite element analysis software.

## 4. FEA Model Definition

The material of the particular harmonic drive components are defined as steel, which is an isotropic material with defined Young’s modulus *E* = 210,000 MPa and the Poisson’s ratio *μ* = 0.3. Each component of the harmonic drive has to be strained just in the elastic range area, where the Hooke’s law can be applied. All contact conditions between the HD components were defined by the Frictionless contact type without the friction consideration (no bonded contact type was used), hence the non-linear analyses were performed.

The parametric model of the harmonic drive unit was created using the most possible relative accuracy of the Creo/Parametric software (1 × 10^−6^). This relative accuracy value allows virtual models to be created that are accurate to ten thousandths of millimeters depending on the maximum dimension of the particular part. This value of the relative accuracy is sufficient enough, because the real production accuracy of the harmonic drive most precise components, e.g., the gearing of the flexspline and circular spline, is manufactured in the range of hundredths of millimeters. The Ansys mesher works with even higher relative accuracy, which guarantees the required level of accuracy also by meshed model.

**Figure 4:**
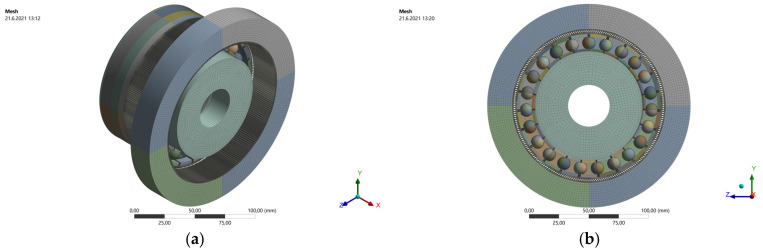

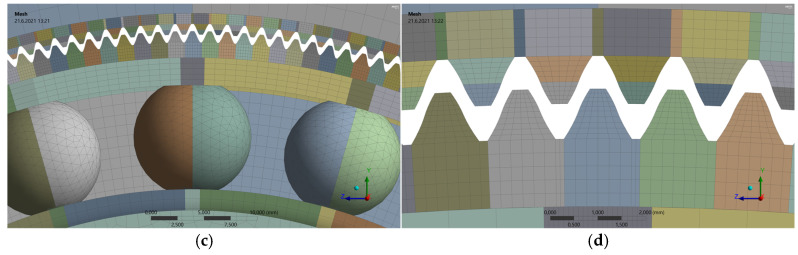
HD 120 FEA model: (**a**) general view; (**b**) front view; (**c**) detail of ball bearing meshing; (**d**) detail of teeth meshing.

The gearing teeth are the most precise elements of the harmonic drive component model. The selected meshing process was performed using the rule to divide each tooth to 10 elements per tooth height and six elements per tooth thickness, independent of the tooth dimension defined in millimeters. In other words, the models of the examined harmonic drive units (HD 42 and HD 120) with the same teeth number have contained the same number of finite elements. The element size of the HD 42 model was approximately 0.035 mm and the element size of the HD 120 model was approximately 0.1 mm as shown in Figure 4.

All HD component boundary conditions were defined by the FEA according to the test procedure conditions described above. The lost motion was evaluated by a test applying the 4% of rated HD output torque to the FS measuring its angular deformation in both directions with the WG locked and the CS fixed and the boundary conditions were adapted to these requirements.

## 5. FEA Analyses Variable Set Generation

The design of virtual experiments was carried out to obtain a representative set of tested parameter combinations. The set of variables used by the analyses of the HD lost motion was generated according the number of chosen input parameters influencing the lost motion and subsequently the torsional backlash by the optimal Space Filling Design method. It is a Latin Hypercube Sampling (advanced form of the Monte Carlo sampling method that avoids clustering samples) optimized to better fill the parameter space. This scheme distributes the design parameters equally throughout the design space, while the corners and/or mid-points are not necessarily included [22]. The method is very useful for the limited computation time, which was the main reason for using it for the presented research.

The parameters influencing the HD lost motion connected with the ball bearing with flexible rings were the following:

1. the bearing inner ring inner diameter *d* (mm);
2. the bearing outer ring outer diameter *D* (mm);
3. the bearing inner radial clearance *G_r_* (mm).

**Figure 5:**
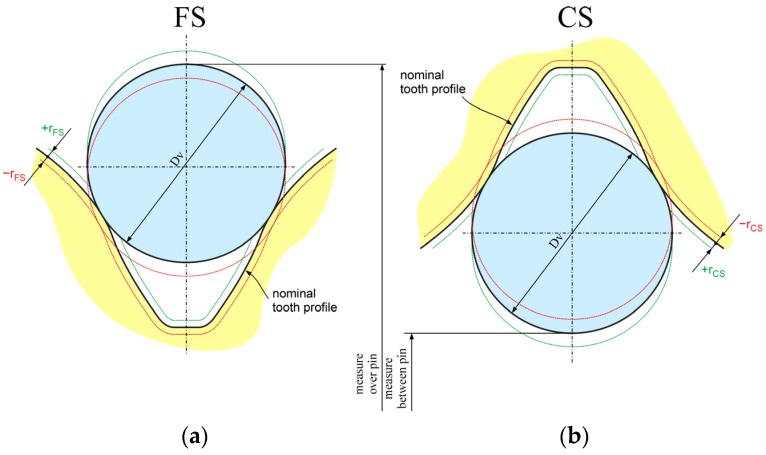
Definition of the offset from the nominal FS and CS teeth shape: (**a**) *r_FS_* influencing the dimension over pin; (**b**) *r_CS_* influencing the dimension between pin.

The parameters influencing the HD backlash connected with the FS and CS teeth spline were following (Figure 5):

4. the offset of the nominal shape of the flexspline tooth *r_FS_* (mm) influencing the dimension over the pin;
5. the offset of the nominal shape of the circular spline tooth *r_CS_* (mm) influencing the dimension between the pin.

The nominal and the limit dimensions of the chosen input parameters are listed in Table 3.

The Ansys workbench software generated, according to the group of five input parameters via the Latin hypercube method, 27 sets of design points for the HD 42, which are listed with the calculated values of the lost motion Φ in Table 4.

The Ansys workbench software generated, according to the group of five input parameters via the Latin hypercube method, 27 sets of design points for the HD 120, which are listed with the calculated values of the lost motion Φ in Table 5.

## 6. Input and Output Variables Correlation Analysis

The parameter correlation performs simulations based on a random sampling of the design space using Latin Hypercube sampling to identify the correlation between all chosen HD parameters. The correlation coefficient indicates whether there is a relationship between the variables and whether the relationship is a positive or a negative number. The closer the correlation coefficient is to the extremes (−1 or 1), the stronger the relationship [23].

The correlation of the input parameters (*d*, *D*, *G_r_*, *r_FS_*, *r_CS_*) and the output parameter (Φ) was observed in the presented study by three methods:

1. Linear regression;
2. Pearson’s linear correlation (detects a linear relationship between two variables);
3. Spearman’s rank correlation (detects a monotonic relationship between two variables).

### 6.1. Linear Regression Model

A linear technique to modeling the relationship between a scalar response and one or more explanatory variables (dependent and independent) is known as linear regression. Simple linear regression is used when there is only one explanatory variable; multiple linear regression is used when there are more than one. Multiple linear regression is a specific example of generic linear models with only one dependent variable, and it is a generalization of simple linear regression with more than one dependent variable. The basic model for multiple linear regression is:

(5) $$Y_{i}=\beta_{0}+\beta_{1}\;X_{i1}+\beta_{2}\;X_{i2}+\ldots +\beta_{p}\;X_{ip}+\varepsilon_{i}$$

for each observation *i* = 1, …, *n*.

In the formula above, there are *n* observations of one dependent variable and *p* independent variables in the calculation above. Thus, *Y_i_* denotes the *i*-th observation of the dependent variable, while *X_ij_* denotes the *i*-th observation of the *j*-th independent variable, with *j* = 1, 2, …, *p*. The values *j* denote the estimated parameters, while *i* is the *i*-th independently identically distributed normal error [24].

### 6.2. Pearson’s Linear Correlation

Pearson’s linear correlation product is the Pearson’s correlation coefficient used to measure the strength of a linear association between two variables, where the value *r* = 1 means a perfect positive correlation and the value *r* = −1 means a perfect negative correlation [25]. Pearson’s correlation coefficient, when applied to a sample, is commonly represented by *r_xy_* and defined as:

(6) $$r_{xy}=\frac{\sum_{i=1}^{n}\left(x_{i}-\bar{x}\right)\left(y_{i}-\bar{y}\right)}{\sqrt{\sum_{i=1}^{n}{\left(x_{i}-\bar{x}\right)}^{2}}\sqrt{\sum_{i=1}^{n}{\left(y_{i}-\bar{y}\right)}^{2}}}$$

where:

*n*—sample size;
*x_i_*, *y_i_*—individual sample points indexed with *i*.

The sample means in the Equation (6) are defined as:

(7) $$\bar{x}=\frac{1}{n}\sum_{i=1}^{n}x_{i}$$

and:

(8) $$\bar{y}=\frac{1}{n}\sum_{i=1}^{n}y_{i}$$

### 6.3. Spearman’s Linear Correlation

The Spearman’s rank correlation coefficient represents a statistical dependence between the rankings of two variables. It determines how well a monotonic function can describe the relationship between two variables. The Pearson correlation between the rank values of two variables is equivalent to the Spearman correlation between those two variables [25].

If all *n* ranks are distinct integers, it can be commonly represented by *r_s_* using the formula:

(9) $$r_{s}=1-\frac{6\sum_{i=1}^{n}d_{i}^{2}}{n\left(n^{2}-1\right)}$$

where:

*n*—sample size (number of observations).

The difference *d_i_* between two ranks (rg) of each observation rg(*X_i_*) and rg(*Y_i_*) is defined as:

(10) $$d_{i}=\mathrm{rg}\left(X_{i}\right)-\mathrm{rg}\left(Y_{i}\right)$$

**Figure 6:**
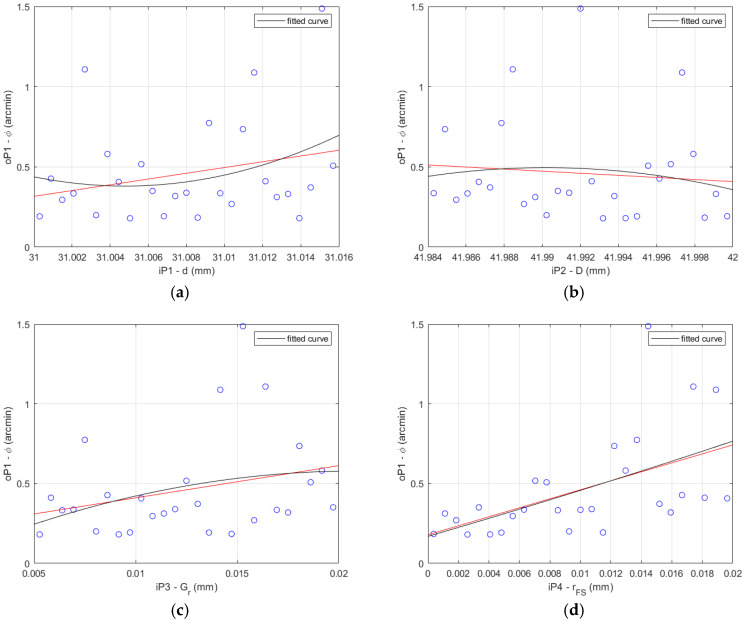

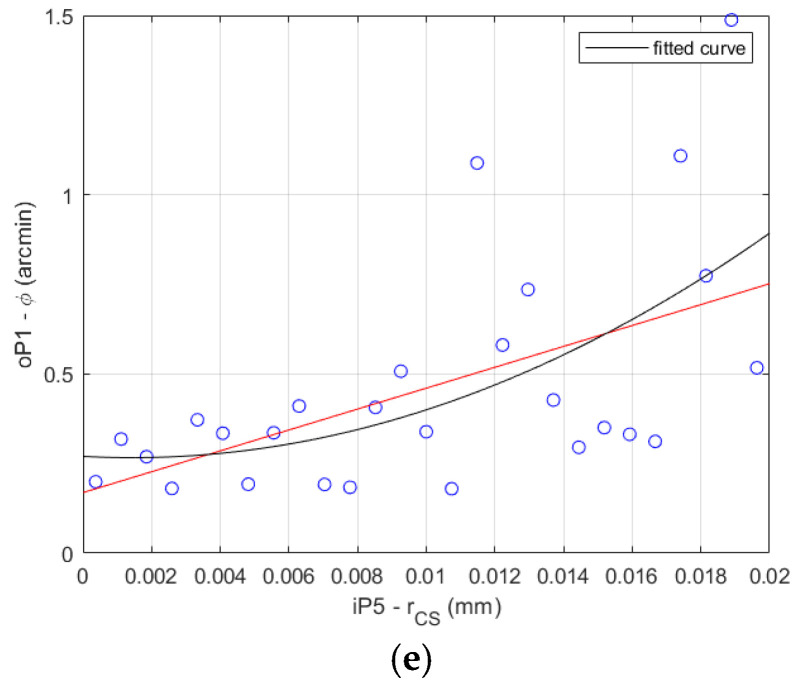
HD 42 determination histograms of the lost motion values (blue circles) applying Pearson (red line) and Spearman (black curve) to the input parameters: (**a**) flexible bearing inner diameter *d*; (**b**) flexible bearing outer diameter *D*; (**c**) flexible bearing radial clearance *G_r_*; (**d**) offset of the FS teeth spline *r_FS_*; (**e**) offset of the CS teeth spline *r_CS_*.

The results of the data correlation applying Pearson and Spearman for the HD 42 are shown in Figure 6.

**Figure 7:**
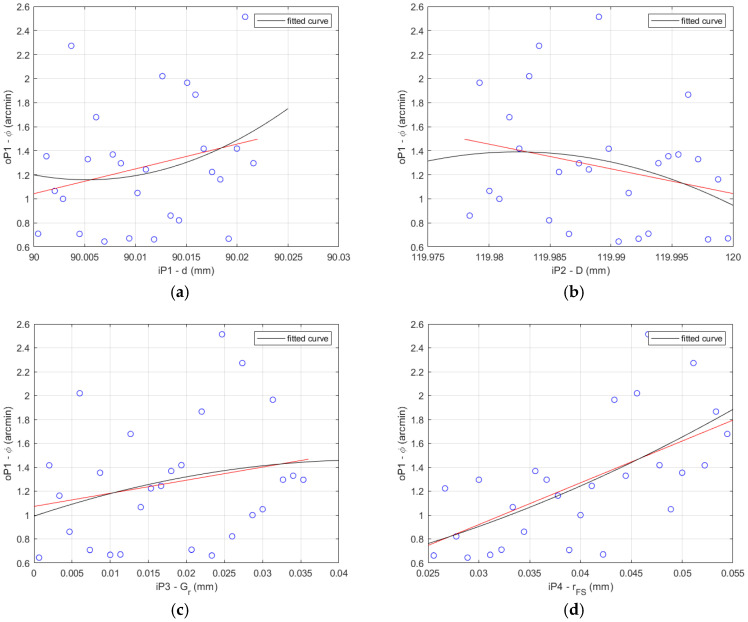

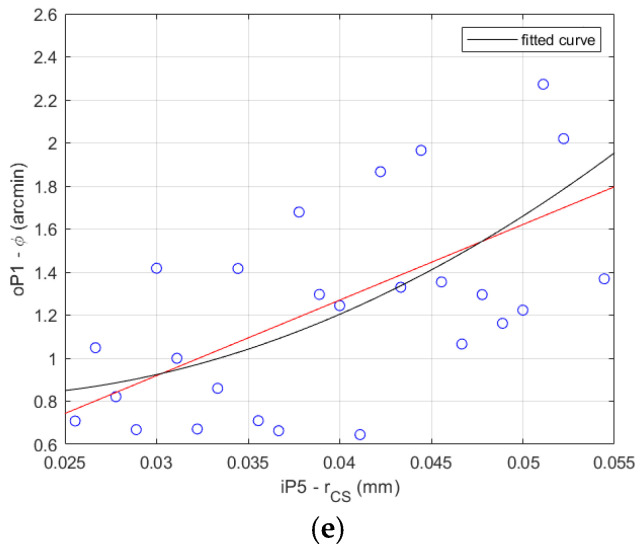
HD 120 determination histograms of the lost motion values (blue circles) applying Pearson (red line) and Spearman (black curve) to the input parameters: (**a**) flexible bearing inner diameter *d*; (**b**) flexible bearing outer diameter *D*; (**c**) flexible bearing radial clearance *G_r_*; (**d**) offset of the FS teeth spline *r_FS_*; (**e**) offset of the CS teeth spline *r_CS_*.

The results of the data correlation applying Pearson and Spearman for the HD 120 are shown in Figure 7.

## 7. Global Sensitivity Analysis

The definition of the global sensitivity analysis (GSA) stated Saltelli et al. [14] as the study of how uncertainty in the output of a model (numerical or otherwise) can be apportioned to different sources of uncertainty in the model input. For this research, the method of conditional variances was used, including the first path and the total effects method.

The global sensitivity analysis of the chosen variables was performed in steps, which were as follows:

1. Input and output dependence polynoma definition;
2. Quasi-random variables data generation;
3. First-order and total-effect Sobol indices calculation.

The second and the third steps were repeated in a cycle until the convergence by the Sobol indices calculation was reached.

### 7.1. Input and Output Dependence Polynoma Definition

The GSA requires the knowledge of the functional dependence between input and output parameters. The whole harmonic drive units FEA models contained approximately 1.5 million elements. The average time demand of one FEA model variant calculation was approximately 24 h. Hence, it was not realistic to calculate thousands of input parameter combination variants. The created polynomial function defining the dependence between the variable input parameters and the harmonic lost motion value was used by the generation of thousands of inputs required for the next calculation of Sobol’s indices.

A second degree polynomial was found using the least squares method, which is described for the lost motion Φ both gearboxes HD 42 and HD 120 for the *n* = 5 chosen parameters by the equation:

(11) $$\Phi =A+\sum_{i=1}^{n}B_{i}\;x_{i}+\sum_{i=1}^{n}\sum_{i>1}^{n}C_{ij}\;x_{i}\;x_{j}+\sum_{i=1}^{n}D_{i}\;x_{i}^{2}$$

where:

A, *B*, *C*, *D*—polynoma coefficents;
*x*—variables (*d*, *D*, *G_r_*, *r_FS_*, *r_CS_*);
*n*—number of variables;
*i* = 1 … *n*—first variable index;
*j* = *i* + 1 … *n*—second variable index.

According the control of the presented considerations, the input data from the FE-analysis data sets were put to the obtained equation and the output results were compared. The quadratic determination correlation coefficient for the presented equation reached the value of R^2^ = 0.98 for HD 42 and R^2^ = 0.99 for HD 120.

**Figure 8:**
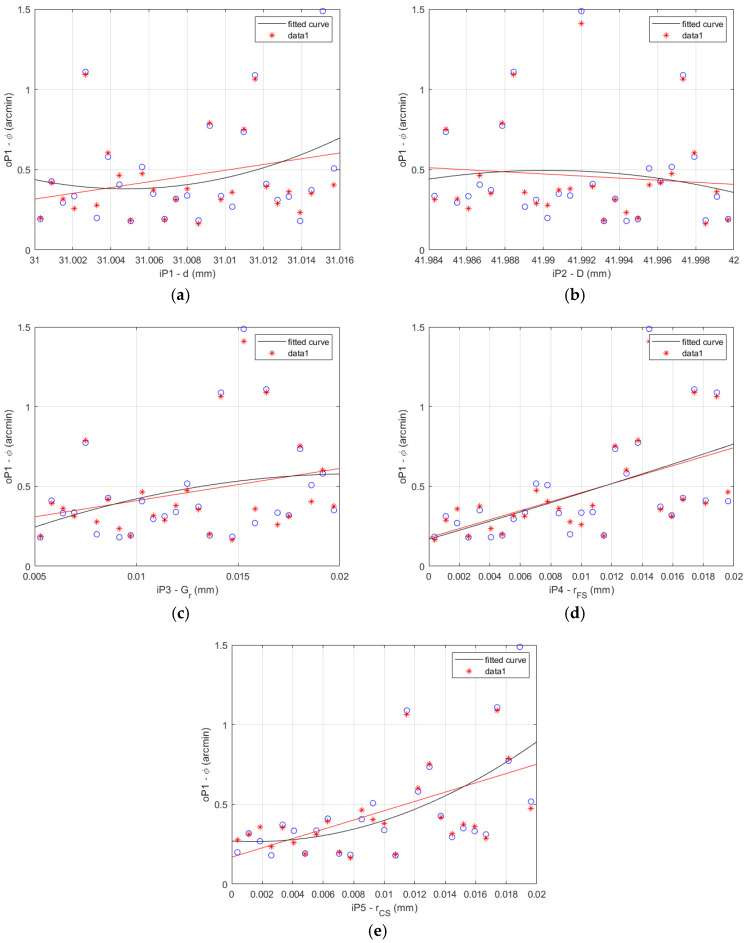
HD 42 lost motion FEA results (blue circles) vs. fitted curve results (red asterisks): (**a**) flexible bearing inner diameter *d*; (**b**) flexible bearing outer diameter *D*; (**c**) flexible bearing radial clearance *G_r_*; (**d**) offset of the FS teeth spline *r_FS_*; (**e**) offset of the CS teeth spline *r_CS_*.

The FEA results and the fitted curve function results comparison is shown for the HD 42 in Figure 8 for the 27 data sets described in Table 4.

**Figure 9:**
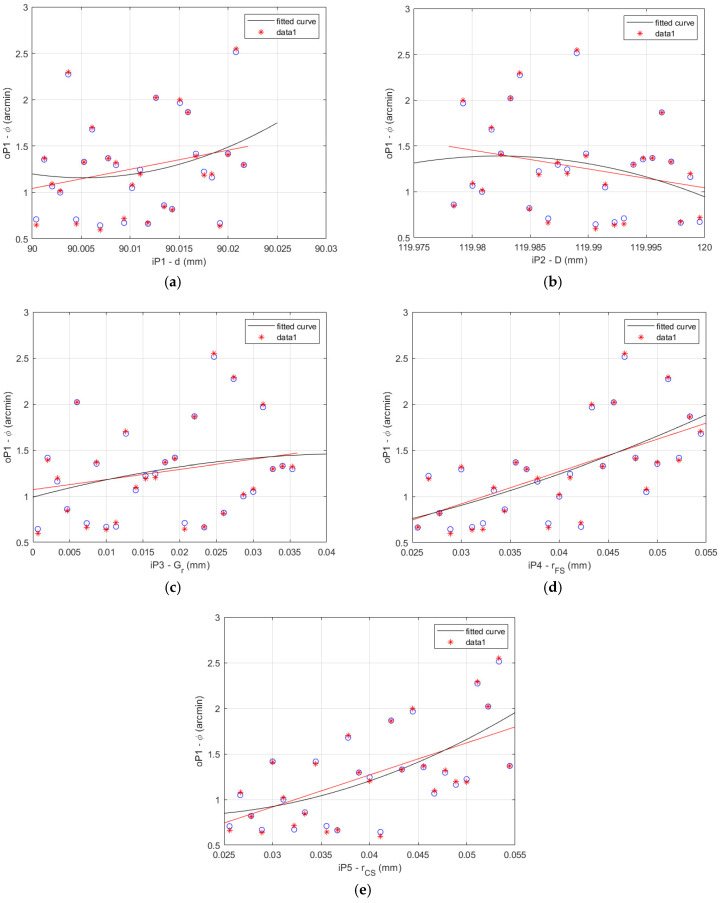
HD 120 lost motion FEA results (blue circles) vs. fitted curve results (red asterisks): (**a**) flexible bearing inner diameter *d*; (**b**) flexible bearing outer diameter *D*; (**c**) flexible bearing radial clearance *G_r_*; (**d**) offset of the FS teeth spline *r_FS_*; (**e**) offset of the CS teeth spline *r_CS_*.

The FEA results and fitted curve function results comparison is shown for the HD 120 in Figure 9 for the 27 data sets described in Table 5.

### 7.2. Quasi-Random Variables Data Generation

Quasi-random variables are not fully random in the sense that they cannot be predicted. An algorithm that generates low-discrepancy sequences must somehow bias the selection of new points to keep them away from the points already existing in order to maintain an even spread of points. They are, nonetheless, similar to random points in that they are uniformly dispersed across the whole variable space as described in [14]. The quasi-random variables can be calculated by various methods generating various data sets. The methods of quasi-random variables data generation used in the presented research contained:

1. Latin Hyper Cube set;
2. Halton set;
3. Sobol set.

The Latin Hyper Cube method has a wide range of uses, but is mostly used in computer engineering. It was invented by McKay et al. from Los Alamos Laboratory in 1979. It has been proven that the Latin Hyper Cube sampling method can increase efficiency compared to Monte Carlo, but this only applies to certain classes of functions [26].

Some quasi Monte Carlo methods work best with specially chosen sample sizes such as large prime numbers or powers of small prime numbers. Halton sequences can be used with any desired sample size. There may be no practical reason to require a richer set of sample sizes than, for example, powers of two. However, first time users may prefer powers of 10, or simply the ability to select any sample size they choose [27].

Sobol sequences are an example of quasi-random sequences. The Russian mathematician Ilya M. Sobol introduced these in 1967. These sequences use a base of two to form successively finer uniform partitions of the unit interval and then reorder the coordinates in each dimension. These sequences are digital (*t*, *s*) sequences in base 2, based on Niederreiter’s former work. The best uniformity of distribution as *n*→∞, good distribution for fairly small initial sets and a very fast computational algorithm are the three main requirements, which are the aim of Sobol sequences [28].

**Figure 10:**
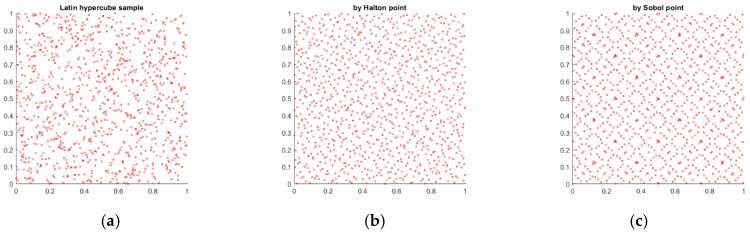
Quasi-random variables scatter plots: (**a**) Latin Hyper Cube set; (**b**) Halton set; (**c**) Sobol set.

The scatter plots of the generated quasi-random variables by the Latin Hyper Cube, Halton and Sobol methods are shown for both gearboxes HD 42 and HD120 in Figure 10.

### 7.3. First-Order and Total-Effect Sobol’s Indices Calculation

Saltelli et al. [14] define the first-order sensitivity index *S_i_* of *X_i_* on *Y* according the equation:

(12) $$S_{i}=\frac{V_{X_{i}}\left(E_{X_{∼i}}\left(Y|X_{i}\right)\right)}{V\left(Y\right)}$$

where the conditional variance:

(13) $$V_{X_{i}}\left(E_{X_{∼i}}\left(Y|X_{i}\right)\right)$$

is called the first-order effect of *X_i_* on *Y*. *S_i_* is a number always between 0 and 1. A high value signals an important variable. Total effects are a direct consequence of Sobol’s variance decomposition approach and estimation procedure. The total effect index accounts for the total contribution to the output variation due to factor *X_i_*, i.e., its first-order effect plus all higher-order effects due to interactions [14].

**Figure 11:**
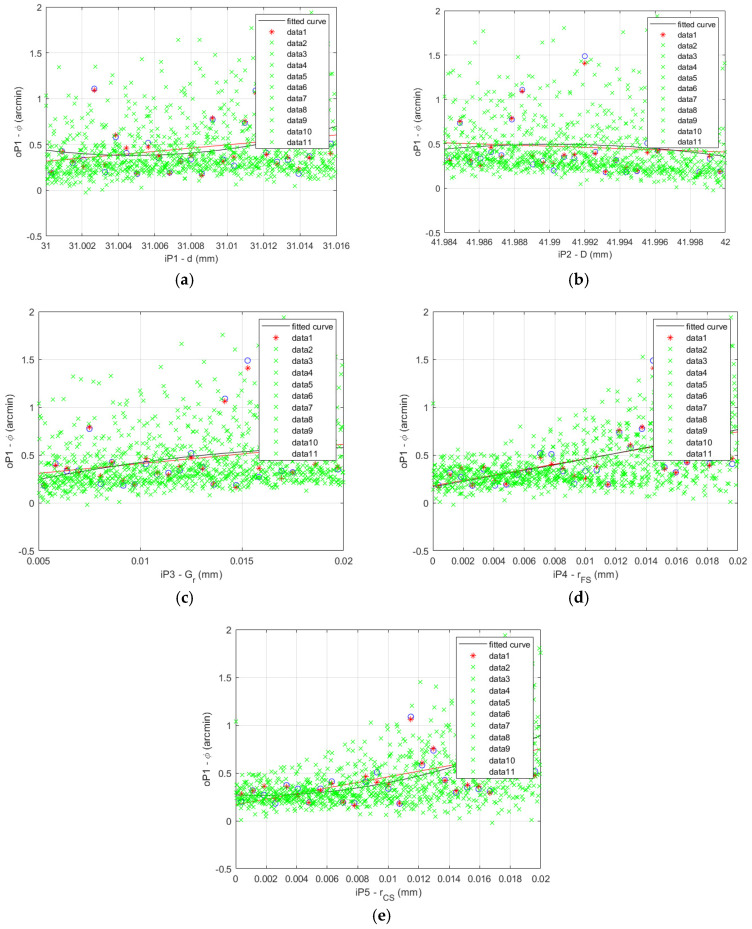
HD 42 lost motion values of the fitted function calculated for the Sobol data set: (**a**) flexible bearing inner diameter *d*; (**b**) flexible bearing outer diameter *D*; (**c**) flexible bearing radial clearance *G_r_*; (**d**) offset of the FS teeth spline *r_FS_*; (**e**) offset of the CS teeth spline *r_CS_*.

The lost motion values of the fitted function calculated for the Sobol data set are shown for the HD 42 in Figure 11.

**Figure 12:**
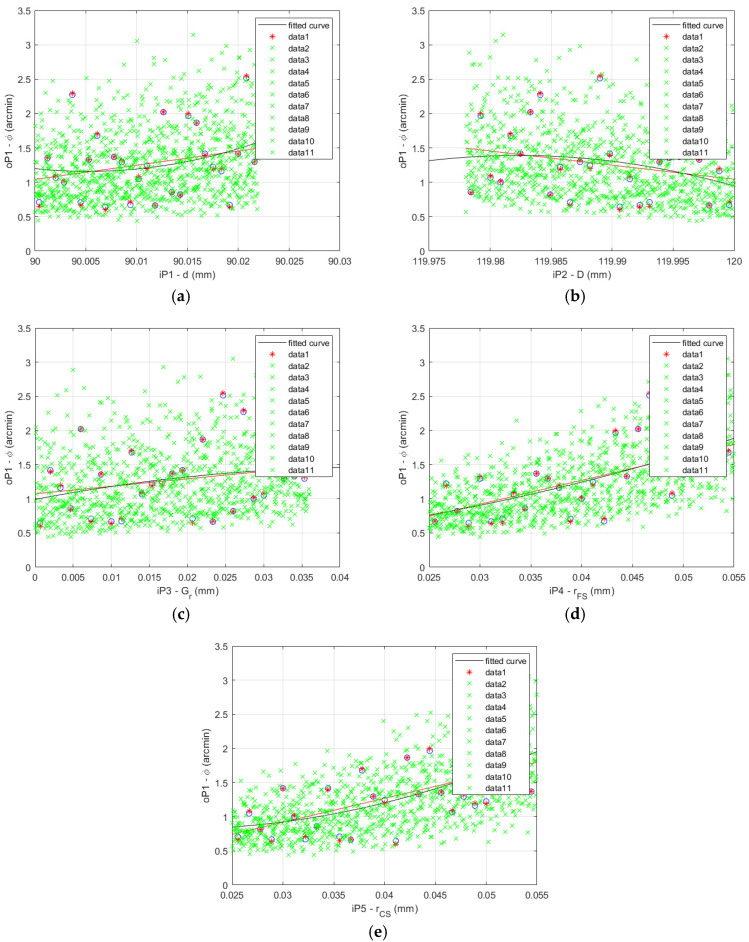
HD 120 lost motion values of the fitted function calculated for the Sobol data set: (**a**) flexible bearing inner diameter *d*; (**b**) flexible bearing outer diameter *D*; (**c**) flexible bearing radial clearance *G_r_*; (**d**) offset of the FS teeth spline *r_FS_*; (**e**) offset of the CS teeth spline *r_CS_*.

The lost motion values of the fitted function calculated for the Sobol data set are shown for the HD 120 in Figure 12.

The data presented in Figure 11 and Figure 12 serve as the base for the calculation of the A, B and C matrices necessary to the Sobol’s indices calculations. The cyclic procedure of their calculation started with a small number of the quasi-random variables, which was gradually increased until the convergence was reached.

**Figure 13:**
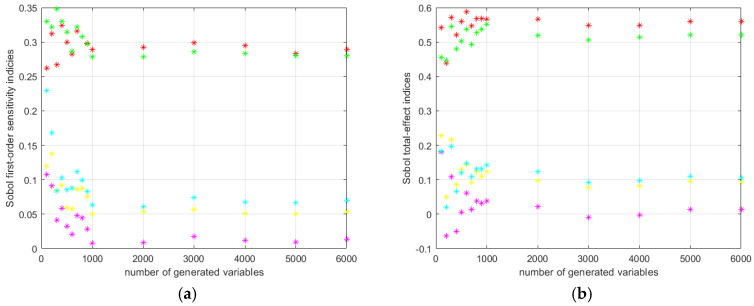
HD 42 Sobol’s indices calculation results: (**a**) first order; (**b**) total effect.

The progress of the cyclic calculation of the first order and total effect Sobol’s indices for the HD 42 are shown in Figure 13.

**Figure 14:**
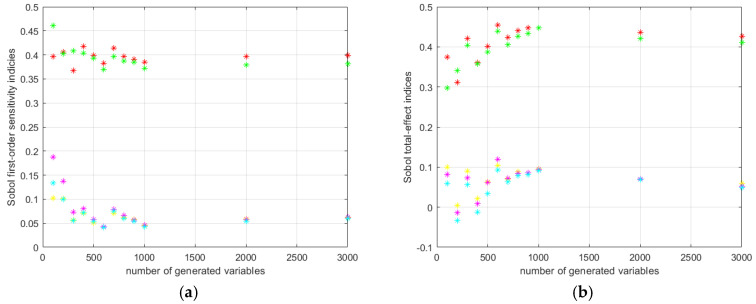
HD 120 Sobol’s indices calculation results: (**a**) first order; (**b**) total effect.

The progress of the cyclic calculation of the first order and total effect Sobol’s indices for the HD 120 are shown in Figure 14.

## 8. Discussion

This research investigated the influence of a change in a five-input parameter set to the value of the harmonic drive (HD) lost motion (torsional backlash) by the prescribed test procedure. The flexspline (FS) of the harmonic drive was loaded by the ±4% of the HD rated output torque by the locked wave generator (WG) and fixed circular spline (CS) and the lost motion value of the flexspline was measured.

**Figure 15:**
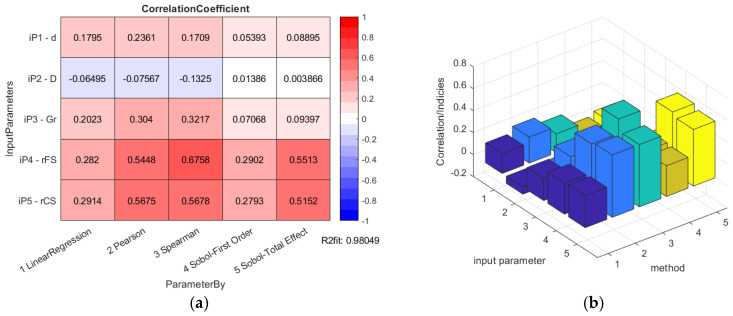
HD 42 global sensitivity analysis calculation results: (**a**) table data; (**b**) column graph.

The overall results of the HD 42 global sensitivity analysis calculations are presented in Figure 15.

**Figure 16:**
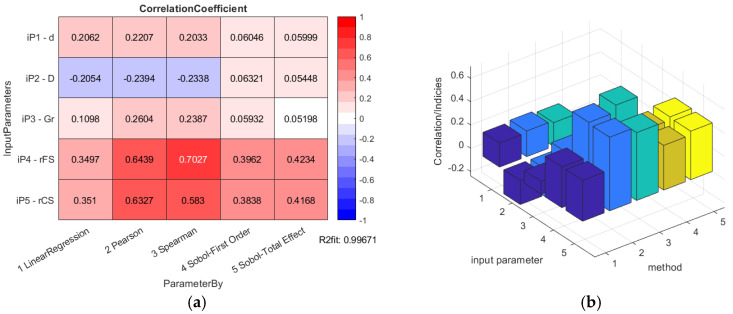
HD 120 global sensitivity analysis calculation results: (**a**) table data; (**b**) column graph.

The overall results of the HD 120 global sensitivity analysis calculations are presented in Figure 16.

Global sensitivity analysis results show the minor impact of the bearing dimensions variations on the value of the HD lost motion, or the torsional backlash, respectively.

The bearing inner diameter *d* was considered to have positive deviations from the nominal dimension simulating the clearance contact between the WG outer shape and the bearing inner ring. The bearing outer diameter *D* was considered with the negative deviations given to the nominal dimension, simulating the clearance between the FS inner shape and the bearing outer ring. The bearing internal diameter *d* has an influence rated by the correlation of about 17.95% to 17.09% by the standard data regression methods for the HD 42, or 20.62% to 20.33% for the HD 120, but this is a minor influence considering the Sobol’s indices values.

The lower the bearing internal radial clearance, the better the deformation of the inner bearing ring on the wave generator outer shape, and the outer bearing ring also matches the FS tube inner surface better. This fact causes a better equidistant match of the WG outer shape and the FS internal surface. The radial clearance of the bearing with flexible rings *G_r_* has an influence rated by the correlation of about 20.23% to 32.17% by the standard data regression methods for the HD 42, or 10.98% to 23.87% for the HD 120, but this is a minor influence considering the Sobol’s indices values.

The higher value of the bearing radial internal clearance causes the imperfection of the component group WG-bearing-FS surfaces in contact with undesirable blank areas without surface contact, especially on the WG minor axis affecting the incorrect FS and CS teeth position accompanied by the teeth interference. The interference, then, occurs not just in the WG minor axis region, but also in the region of the teeth contact path start and end inclined from the WG minor axis at the angle of approximately 60° in both directions.

The bearing parameter group interconnectedness can cause the accumulation of their effect on the WG-bearing-FS surface match accuracy, and also their effect on the lost motion according to the parameter combination. The same value of the bearing internal clearance by various values of the bearing diameters causes significantly different impacts to the WG-bearing-FS surface match and also the lost motion. The various bearing parameter sets cause various influence to the HD lost motion and subsequently the torsional backlash value, which has an impact on the Sobol’s indices values. The parameters of the bearing with flexible rings (*d*, *D* and *G_r_*) are intertwined, which influences the lower coefficients by the outer bearing ring diameter *D* for the HD 42 and similar values to the inner bearing diameter *d* for the HD 120.

The total-effect Sobol’s indices of the bearing parameter set for the HD 120 are similar (0.05999; 0.05448; 0.05198), because all of them affect the bearing radial clearance and subsequently the HD component surface match and also the lost motion value in the same way. The total-effect Sobol’s indices of the bearing parameter set for the HD 42 are different from the ones calculated for the HD 120. This is caused by the variation in the chosen ISO tolerance grade IT6, defined for various HD nominal dimensions (0.016 mm for the HD 42 versus 0.022 mm for the HD 120) which generates various bearing clearance values (0.02 mm for the HD 42 versus 0.036 mm for the HD 120).

## 9. Conclusions

Global sensitivity analysis by both HD variants let to the same parameter affecting the lost motion, and subsequently the torsional backlash the most; the offsets from the nominal FS and CS teeth shape *r_FS_* and *r_CS_*. They have the largest size among the observed parameters group and thus their criteria weight is highest too. Their Sobol’s indices values are close to each other. The change in *r_FS_* and *r_CS_* affects the dimension over pin for the FS and between pin for the CS, and also the lost motion in the same way; equal changes in the offsets generate equal change in the output parameter, that is, the HD lost motion.

The highest correlation is observed for the HD 42 by the Spearman’s rank correlation (67.58%), but the Sobol’s indices total effect method is also pointing out a high level of correlation between 51.52% (FS offset), or the 55.13% (CS offset), respectively. The first order Sobol’s indices show just the correlation of 29.02% (FS offset) to 27.93% (CS offset), respectively. The highest correlation is observed for the HD 120 by the Spearman’s rank correlation (70.27%), but the total effect Sobol’s indices also show a high level of correlation of 42.34% (FS offset), or 41.68% (CS offset), respectively. The first order Sobol’s indices show just the correlation of 39.62% (FS offset) to 38.38% (CS offset), respectively.

The different values of the Sobol’s indices found with the different offsets are the consequence of the various diameters of the examined harmonic drive units. They have the same teeth number and gear ratio, but the modules are quite different: the tooth height of the HD 42 is only 0.337 mm, while the tooth height of the HD 120 is 1 mm. The flexspline thickness affecting the torsional stiffness is also different for both HD units and thus, the values of the lost motion performed by the FEA of the HD 42 and HD 120 are not equal, which has also impact to the different values of the Sobol’s indices.

The HD teeth spline offset from the nominal shape has the highest impact to the HD lost motion value by both virtual tested units. The proper selection of the *r_FS_* and *r_CS_* values will lead to the required low value of the HD backlash during its operation in the desired motion state.

## Figures and Tables

**Table 1:** Harmonic drive HD 42 and HD 120 parameters.

HD type	*z_FS_* (-)	*z_CS_* (-)	*I* (-)	*n*_1_ (min^−1^)	*P*_1_ (W)	4% (of *T_N_*) (N m)
HD 42	240	242	−120	1500	31	1 (of 24)
HD 120	240	242	−120	1500	693	21 (of 529)

**Table 2:** Nominal dimensions of the PBF standard ball bearings with flexible rings.

Bearing Type	*d* (mm)	*d*_1*max*_ (mm)	*D*_1*min*_ (mm)	*D* (mm)	*B* (mm)	*O* (mm)
113-1145TN	31.00	33.10	40.10	42.00	7.00	0.30–0.56
114-876TN	90.00	95.20	114.80	120.00	18.00	1.20–1.60

**Table 3:** Limit input dimensions of the chosen HD parameter set for the Space Filling Design method.

Limit Dimensions		*d* (mm)	*D* (mm)	*G_r_* (mm)	*r_FS_* (mm)	*r_CS_* (mm)
HD 42	nominal	31.000	42.000	0.015	0.000	0.000
	minimal	31.000	41.984	0.005	0.000	0.000
	maximal	31.016	42.000	0.020	0.020	0.020
HD 120	nominal	90.000	120.000	0.030	0.025	0.025
	minimal	90.000	119.978	0.000	0.025	0.025
	maximal	90.022	120.000	0.055	0.055	0.055

**Table 4:** Variable sets generated for the HD 42 by the Space Filling Design method with calculated lost motion values.

Data Set	*d* (mm)	*D* (mm)	*G_r_* (mm)	*r_FS_* (mm)	*r_CS_* (mm)	Φ (arcmin)
1	31.0080000000	41.9914074074	0.0119444444	0.0107407407	0.0100000000	0.3387054552
2	31.0068148148	41.9997037037	0.0097222222	0.0114814815	0.0048148148	0.1924051335
3	31.0020740741	41.9860740741	0.0169444444	0.0100000000	0.0040740741	0.3345637636
4	31.0115555556	41.9973333333	0.0141666667	0.0188888889	0.0114814815	1.0883206873
5	31.0032592593	41.9902222222	0.0080555556	0.0092592593	0.0003703704	0.1993212189
6	31.0074074074	41.9937777778	0.0175000000	0.0159259259	0.0011111111	0.3183620597
7	31.0056296296	41.9967407407	0.0125000000	0.0070370370	0.0196296296	0.5170704345
8	31.0151111111	41.9920000000	0.0152777778	0.0144444444	0.0188888889	1.4873940404
9	31.0044444444	41.9866666667	0.0102777778	0.0196296296	0.0085185185	0.4067272251
10	31.0038518519	41.9979259259	0.0191666667	0.0129629630	0.0122222222	0.5806961197
11	31.0050370370	41.9931851852	0.0052777778	0.0025925926	0.0107407407	0.1798381857
12	31.0062222222	41.9908148148	0.0197222222	0.0033333333	0.0151851852	0.3502783652
13	31.0002962963	41.9949629630	0.0136111111	0.0048148148	0.0070370370	0.1918537718
14	31.0109629630	41.9848888889	0.0180555556	0.0122222222	0.0129629630	0.7353834863
15	31.0091851852	41.9878518519	0.0075000000	0.0137037037	0.0181481481	0.7737438484
16	31.0103703704	41.9890370370	0.0158333333	0.0018518519	0.0018518519	0.2691275760
17	31.0145185185	41.9872592593	0.0130555556	0.0151851852	0.0033333333	0.3720114037
18	31.0157037037	41.9955555556	0.0186111111	0.0077777778	0.0092592593	0.5075938888
19	31.0014814815	41.9854814815	0.0108333333	0.0055555556	0.0144444444	0.2953603605
20	31.0008888889	41.9961481481	0.0086111111	0.0166666667	0.0137037037	0.4271771907
21	31.0139259259	41.9943703704	0.0091666667	0.0040740741	0.0025925926	0.1805950189
22	31.0127407407	41.9896296296	0.0113888889	0.0011111111	0.0166666667	0.3117640981
23	31.0121481481	41.9925925926	0.0058333333	0.0181481481	0.0062962963	0.4104898389
24	31.0097777778	41.9842962963	0.0069444444	0.0062962963	0.0055555556	0.3359137403
25	31.0133333333	41.9991111111	0.0063888889	0.0085185185	0.0159259259	0.3315779483
26	31.0026666667	41.9884444444	0.0163888889	0.0174074074	0.0174074074	1.1083372325
27	31.0085925926	41.9985185185	0.0147222222	0.0003703704	0.0077777778	0.1838389297

**Table 5:** Variable sets generated for the HD 120 by the Space Filling Design method with calculated lost motion values.

Data Set	*d* (mm)	*D* (mm)	*G_r_* (mm)	*r_FS_* (mm)	*r_CS_* (mm)	Φ (arcmin)
1	90.011000000	119.988185185	0.016666667	0.041111111	0.040000000	1.244209085
2	90.009370370	119.999592593	0.011333333	0.042222222	0.032222222	0.671159038
3	90.002851852	119.980851852	0.028666667	0.040000000	0.031111111	0.999485343
4	90.015888889	119.996333333	0.022000000	0.053333333	0.042222222	1.866310632
5	90.004481481	119.986555556	0.007333333	0.038888889	0.025555556	0.708068406
6	90.010185185	119.991444444	0.030000000	0.048888889	0.026666667	1.048538552
7	90.007740741	119.995518519	0.018000000	0.035555556	0.054444444	1.368646926
8	90.020777778	119.989000000	0.024666667	0.046666667	0.053333333	2.513551579
9	90.006111111	119.981666667	0.012666667	0.054444444	0.037777778	1.678786696
10	90.005296296	119.997148148	0.034000000	0.044444444	0.043333333	1.329323399
11	90.006925926	119.990629630	0.000666667	0.028888889	0.041111111	0.644800974
12	90.008555556	119.987370370	0.035333333	0.030000000	0.047777778	1.295404011
13	90.000407407	119.993074074	0.020666667	0.032222222	0.035555556	0.709814782
14	90.015074074	119.979222222	0.031333333	0.043333333	0.044444444	1.965452675
15	90.012629630	119.983296296	0.006000000	0.045555556	0.052222222	2.019798297
16	90.014259259	119.984925926	0.026000000	0.027777778	0.027777778	0.821133320
17	90.019962963	119.982481481	0.019333333	0.047777778	0.030000000	1.417778345
18	90.021592593	119.993888889	0.032666667	0.036666667	0.038888889	1.296064918
19	90.002037037	119.980037037	0.014000000	0.033333333	0.046666667	1.065814949
20	90.001222222	119.994703704	0.008666667	0.050000000	0.045555556	1.354223000
21	90.019148148	119.992259259	0.010000000	0.031111111	0.028888889	0.667895757
22	90.017518519	119.985740741	0.015333333	0.026666667	0.050000000	1.223274066
23	90.016703704	119.989814815	0.002000000	0.052222222	0.034444444	1.416564820
24	90.013444444	119.978407407	0.004666667	0.034444444	0.033333333	0.860107056
25	90.018333333	119.998777778	0.003333333	0.037777778	0.048888889	1.161955836
26	90.003666667	119.984111111	0.027333333	0.051111111	0.051111111	2.272618801
27	90.011814815	119.997962963	0.023333333	0.025555556	0.036666667	0.662908446

## Data Availability

Not applicable.
